# Electrosensitive Spatial Vectors in Elasmobranch Fishes: Implications for Source Localization

**DOI:** 10.1371/journal.pone.0016008

**Published:** 2011-01-13

**Authors:** Ariel C. Rivera-Vicente, Josiah Sewell, Timothy C. Tricas

**Affiliations:** 1 Department of Zoology, University of Hawaii at Manoa, Honolulu, Hawaii, United States of America; 2 The Hawaii Institute of Marine Biology, Kaneohe, Hawaii, United States of America; University of California Davis, United States of America

## Abstract

The electrosense of sharks and rays is used to detect weak dipole-like bioelectric fields of prey, mates and predators, and several models propose a use for the detection of streaming ocean currents and swimming-induced fields for geomagnetic orientation. We assessed pore distributions, canal vectors, complementarity and possible evolutionary divergent functions for ampullary clusters in two sharks, the scalloped hammerhead (*Sphyrna lewini)* and the sandbar shark (*Carcharhinus plumbeus*), and the brown stingray (*Dasyatis lata*). Canal projections were determined from measured coordinates of each electrosensory pore and corresponding ampulla relative to the body axis. These species share three ampullary groups: the buccal (BUC), mandibular (MAN) and superficial ophthalmic (SO), which is subdivided into anterior (SOa) and posterior (SOp) in sharks. The stingray also has a hyoid (HYO) cluster. The SOp in both sharks contains the longest (most sensitive) canals with main projections in the posterior-lateral quadrants of the horizontal plane. In contrast, stingray SO canals are few and short with the posterior-lateral projections subsumed by the HYO. There was strong projection coincidence by BUC and SOp canals in the posterior lateral quadrant of the hammerhead shark, and laterally among the stingray BUC and HYO. The shark SOa and stingray SO and BUC contain short canals located anterior to the mouth for detection of prey at close distance. The MAN canals of all species project in anterior or posterior directions behind the mouth and likely coordinate prey capture. Vertical elevation was greatest in the BUC of the sandbar shark, restricted by the hammerhead cephalofoil and extremely limited in the dorsoventrally flattened stingray. These results are consistent with the functional subunit hypothesis that predicts specialized ampullary functions for processing of weak dipole and geomagnetic induced fields, and provides an anatomical basis for future experiments on central processing of different forms of relevant electric stimuli.

## Introduction

The transduction and encoding of directional information from an external stimulus is critical for localization of other organisms and environmental cues. The identification and location of a target can involve different processing of stimuli that originate from a visual [1], chemical [2], [3] or acoustic [4]–[6] target. Some organisms such as bats actively query the environment with self-generated acoustic pulses [7], [8] or electric stimuli as in some teleost fish [9], [10]. These studies show that great variation exists in the peripheral and central mechanisms for extraction of important target features such as direction, azimuth, elevation, velocity or size that are used for related orientation behaviors.

The ampullary electroreceptors of elasmobranch fishes (sharks, skates and rays) are unique sensory organ arrays that mediate detection and localization of weak electric stimuli. The functional electrosensory unit is the ampulla of Lorenzini which consists of a small subdermal receptor chamber that is connected by a narrow canal (∼1 mm diam) to a single pore on the skin. Both the ampullary chamber and canal are filled with a continuous ion-rich hydrogel that has similar dc conductivity but different electrical admittance properties than seawater that contribute to frequency response properties [11]–[13]. The receptor cells function as voltage detectors in the lumen and encode the potential difference between the apical membrane in the lumen and the basal membrane in the surrounding extra-ampullary tissues [14]. These stimuli control release of neurotransmitter that modulates the discharge rates of primary afferent neurons that convey neural codes to the brain. Neurophysiology experiments show primary afferents are most sensitive to varying electric fields at low frequencies from 0.1–10 Hz with threshold sensitivity to uniform electric field stimuli as low as 20 ηV/cm [15]–[18]. Behavioral experiments demonstrate lower orientation thresholds of 2 ηV/cm [19].

Electric fields of biological relevance in aquatic environments arise from animate and inanimate sources [20]. Behavioral studies show that elasmobranchs detect weak bioelectric fields produced by prey [21], [22], mates [23] and predators [24]. Dominant dc electric fields from these living organisms result from the separation of ionic charges in the body and can be modeled as a *dipole* electric field at distance. In contrast, stimuli from non-living sources include electric fields that may arise from interactions with the Earth's magnetic field [20], [25]. It is proposed that oceanic and tidal currents that stream through the vertical component of the Earth's magnetic field produce horizontal *uniform* electric fields that could be detected and used to perceive drift by electrosensitive fish. The relatively constant direction of these fields may allow animals drifting in the ocean to maintain a constant heading relative to the water current stream (passive mode) [25] but direct empirical tests are few [26]. In addition, it is proposed that a shark that swims through the Earth's magnetic field induces an orthogonal electric field across its head and body (active mode) from which the polarity and intensity of the induced vertical electric field can be discriminated. Another hypothesis proposes that electric fields induced by locomotor movements should be detected by vertically oriented ampullary canals and centrally integrated with horizontal vestibular information to provide a compass sense [27].

**Figure 1:**
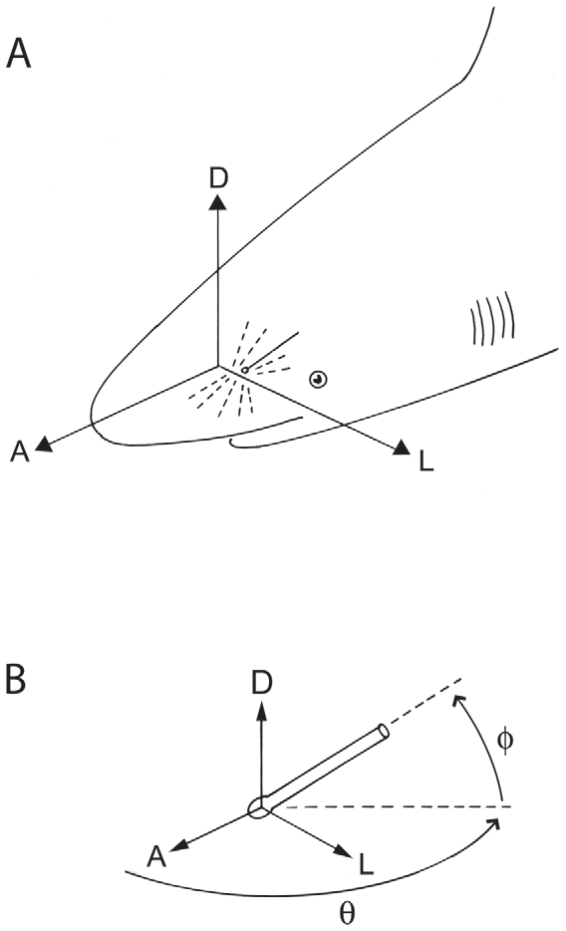
Directions of electrosensory canal projections relative to the shark body. (A) Projections originate along the central body axis and are anterior (A), dorsal (D) or lateral (L) relative to the body, with complementary posterior, ventral and medial projections, respectively (not illustrated). (B) Spherical projection vectors for each ampullary canal are expressed as direction relative to the shark body. Direction origins are at the ampulla and have projections relative to the anterior, dorsal or lateral direction of the body. Azimuth (θ, *theta)* is calculated as the angle of deviation from the anterior direction in the horizontal plane, and elevation (ϕ, *phi*) in the orthogonal vertical plane.

In marine sharks and rays the ampullae are organized into distinct subdermal groups or clusters that are associated with branches of the anterior lateral line nerve [28] to guide orientation behaviors. The ampullary canals radiate in different directions from these clusters to pores distributed widely over the head (and body of batoids) (Fig. 1). The spatial separation of each ampulla and its pore results in canal projections that are multidirectional with respect to the body axis of the animal and surrounding space. The spatial arrangement of the electrosensory array is an important determinant for localization of electric field sources, but the translation of complex field stimuli by the entire array complex in space is uncharacterized. Sharks may follow electric field lines from an external source by maintaining a constant representation of the field signature on the head, or alternatively may derive the source location by differential sampling across the electrosensory array [29]. Detailed studies of the orientation patterns to dipole electric stimuli show that there is great variation in approach paths of some sharks [30]. The somatotopic representation of these external fields results from characteristics of the array such as the number and position of clusters, number of ampullary canals, and the length and spatial projection of canals relative to the midline of the animal. For example, ampullae with long canals will sample a larger segment of a *uniform* electric field and their associated primary afferent neurons will receive proportionally more excitation (or inhibition) than ampullae with shorter canals. In addition, canals that are oriented parallel to *uniform* field lines will be maximally excited and those with orthogonal orientation will be insensitive [15], [31]. Recent work shows that the spatial projections of the skate hyoid array in two dimensions can enhance coding efficiency by the peripheral nervous system for a dipole stimulus [32]. Thus, characterization of the spatial organization of the electrosensory array is needed to develop realistic peripheral and central neural computation models.

Several studies describe the spatial arrangement for hundreds or thousands of electrosensory pores in different elasmobranch species [33]–[39] but most functional analyses are limited to assessment of dorsal and ventral surface pore distributions in relation mainly to their feeding ecology. One study has examined the spatial projections of canal arrays in the white shark and horizontal projections in the barn door skate [40]. However, one limitation of that study is that ampullae of each cluster in the shark were modeled as originating from a common central reference point, thus absolute canal projection data could not be reported. In addition, vectors were presented in orthogonal Cartesian coordinates so projection vectors in spherical coordinates are not known. Nonetheless, that work showed discrete differences in the planar projections of ampullary subgroups and proposed the functional subunit hypothesis that states the ampullary arrays are divided into morphologically distinct groups or subgroups and serve different primary functions such as orientation to prey and processing of uniform electric fields [40].

The present study compares the projection vectors of individual ampullary canals from cluster arrays in three divergent elasmobranch species. The carcharhinid sandbar shark (*Carcharhinus plumbeus*) has a pseudo-conical head and is wide-ranging in coastal temperate and sub-temperate seas. The hammerhead shark (*Sphyrna lewini*), a large coastal species in tropical and subtropical waters, is derived from a common ancestor of the carcharhinid sharks and has evolved a dorsoventrally flattened head (cephalofoil). The brown stingray (*Dasyatis lata*) is a derived batoid that shows extreme dorsoventral compression of the head and body. In this analysis, we examine several predictions derived from the functional subunit hypothesis: 1) evolutionary divergence or convergence of canal projection patterns in 3D space, 2) overlap or complementarity in directional patterns among and within cluster groups, and 3) potential loss of sensitivity to vertical fields that is associated with the dorsal-ventral compression of the head and body. This work shows that bilaterally symmetrical ampullary arrays form an asymmetrical 3D directional antenna that may compare intensity differences and encode directional information from complex electric stimuli. We show several similarities and differences among spatial projection patterns for the main ampullary clusters, and quantify the constraint of a dorsoventrally flattened body on sampling of vertical electric field stimuli. These features permit identification of specific ampullary groups or subgroups likely to be involved in encoding and central processing of electric stimuli and guidance of subsequent orientation behaviors to biotic and abiotic electric sources.

## Results

### Array morphology

The adult hammerhead shark had a total of 1362 ampullae on the left side of the head and the greatest number for any of our study species, with 45% of pores on the dorsal surface of the head and 55% of pores on the ventral surface (Table 1). The estimated total number of ampullae reported here (2720) for the adult hammerhead is slightly less than that previously reported for juveniles (total of 2796–3400 pores)[34]. This difference may reflect natural variation among individuals or perhaps inclusion of pit organ pores in the juvenile specimens. In comparison, the adult sandbar shark specimen had 1021 on the left side of the head ampullae (estimated 2041 total) with 48% projecting to pores on the dorsal and 52% to pores on the ventral surfaces. Our data for *C. plumbeus* fall within the range previously reported [34], [39]. The dorsoventrally flattened stingray had 743 canals on the left side of the head (estimated 1486 total) of which 85% projected to the ventral surface. The number and predominantly ventral projection of canals in the stingray is in accord with previous reports from several benthic skate species [36], [40].

**Table 1:** Ampullary canals in the dorsal and ventral groups of ampullary clusters on the left side of the head in the sandbar shark (*Carcharhinus plumbeus*), scalloped hammerhead shark (*Sphyrna lewini*) and brown stingray (*Dasyatis lata*).

	BUC		SO		HYO		MAN
	D	V	D	V	D	V	V
Sandbar Shark			SOa	SOa			
	n = 167	n = 114	n = 157	n = 270	*np*	*np*	n = 6
	4.55±1.57	4.62±2.68	4.23±1.05	3.52±0.54			6.27±1.96
	1.85–8.15	0.57–11.74	1.81–6.35	1.84–4.65			3.92–8.44
			SOp	SOp			
			n = 170	n = 137			
			8.31±3.21	3.27±1.15			
			2.82–16.11	1.17–6.58			
Hammerhead Shark			SOa	SOa			
	n = 186	n = 111	n = 310	n = 464	*np*	*np*	n = 7
	3.97±2.54	4.97±1.63	4.38±2.23	4.85±1.51			2.73±1.49
	1.10–9.30	2.58–7.20	1.82–11.95	3.07–12.15			1.17–5.47
			SOp	SOp			
			n = 117	n = 167			
			8.63±2.36	5.90±1.87			
			3.99–15.23	1.56–9.68			
Stingray	n = 11	n = 178	n = 16	n = 55	n = 87	n = 380	n = 16
	5.29±0.84	3.41±2.03	1.19±0.37	2.82±2.17	8.32±2.73	5.08±3.45	1.25±0.3
	4.17–6.80	0.37–9.53	0.71–1.75	0.17–7.32	2.76–13.98	0.14–22.22	10.71–.71

Number of canals per cluster (n), mean ± SD and ranges of canal lengths (cm) are shown. BUC  =  buccal, HYO  =  hyoid, SO  =  superficial ophthalmic, SOa  =  superficial ophthalmic anterior, SOp  =  superficial ophthalmic posterior, MAN  =  mandibular. Hyoid ampullae are not present (*np*) in the two species of shark.

**Figure 2:**
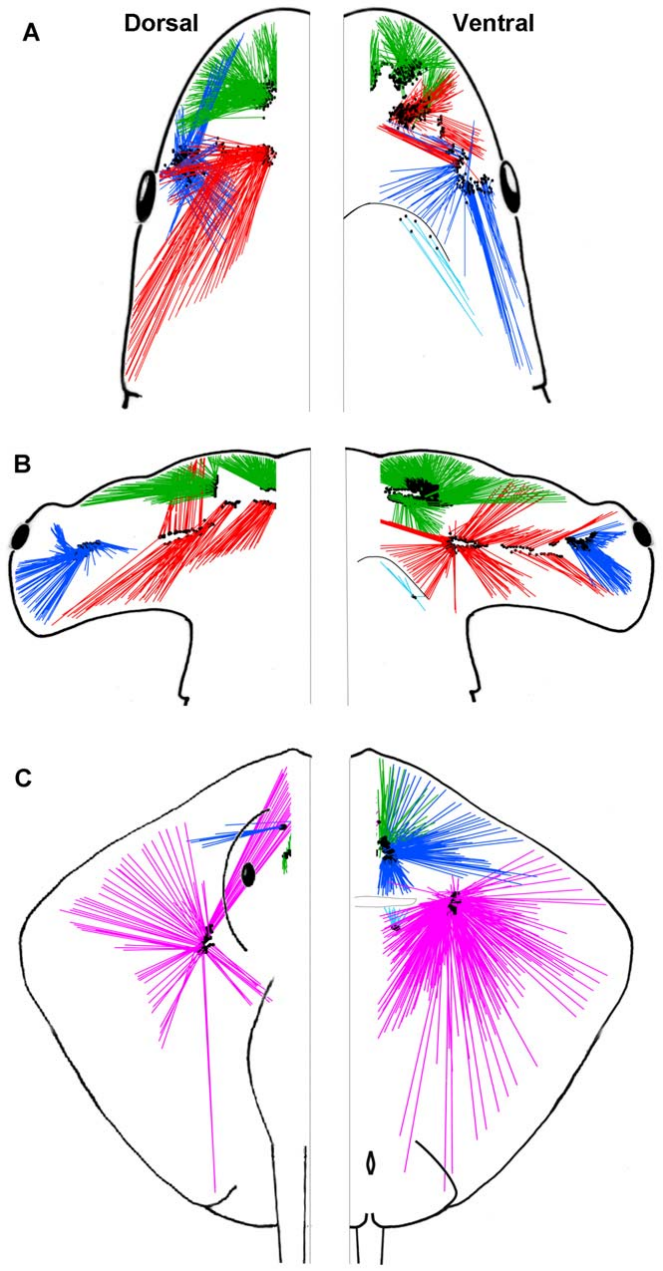
Horizontal view of the electrosensory arrays of the sandbar shark, *Carcharhinus plumbeus*. (A), scalloped hammerhead shark, *Sphyrna lewini* (B) and brown stingray, *Dasyatis lata* (C). Canals with pores on the dorsal and ventral surface are shown on the left and right side of the figure, respectively. Canals from each ampullary group are represented by different colors (BUC  =  blue, SOa  =  green, SOp  =  red, HYO  =  pink). Location of ampullae are indicated by black dots at the base of canals.

The electrosensory ampullae on the left side of the head in the hammerhead and sandbar sharks were classified into three clusters that followed the branch of the associated anterior lateral line nerve: the buccal (BUC), superficial ophthalmic (SO) and mandibular (MAN). The SO nerve further divides into branches that innervate the physically distinct superficial ophthalmic anterior (SOa) and superficial ophthalmic posterior (SOp) sub-clusters (Fig. 2A, 2B). In the stingray, four distinct clusters are present: the BUC, the hyoid (HYO), the SO, and the MAN (Fig. 2C). In all species, the MAN cluster only projects to the ventral surface, whereas all other ampullary clusters had canal projections to both the dorsal and ventral surfaces of the head.

### Cluster projection vectors

#### Sandbar shark

**Figure 3:**
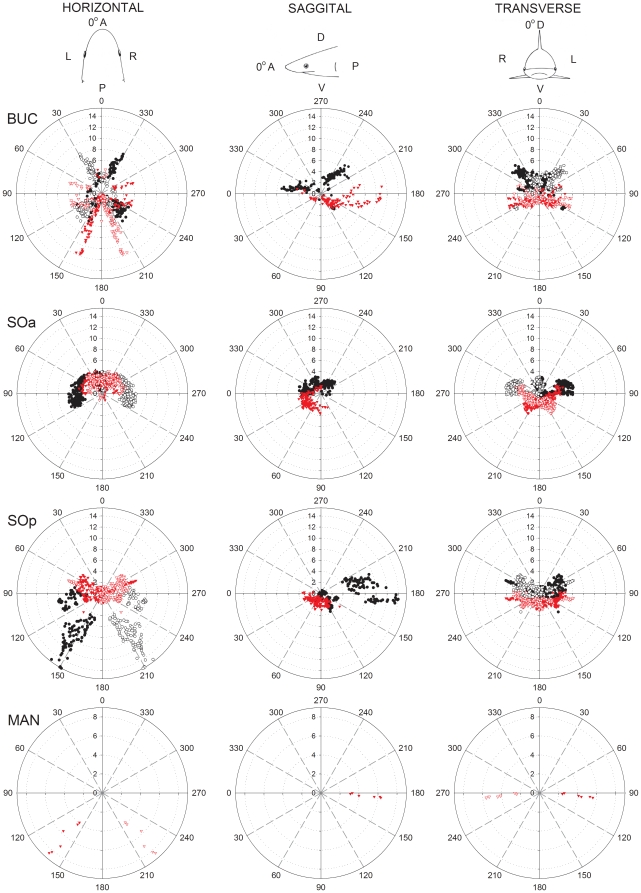
Polar plot of canal projections in three planes of the electrosensory ampullary array of the sandbar shark, *Carcharhinus plumbeus*. Plots show canal length (cm) vs. canal orientation angle in the horizontal, sagittal and transverse planes for each ampulla group (BUC  =  buccal; SOa  =  superficial ophthalmic anterior; SOp  =  superficial ophthalmic posterior; MAN  =  mandibular). Projections of ventral and dorsal canals are indicated with red and black symbols, respectively. Projections are shown for both left (filled symbols) and right (open symbols) ampullary clusters. Reference directions for each plane are *horizontal*: 0° =  anterior (A), 90° =  left (L), 180° =  posterior (P), 270° =  right (R); *sagittal* : 0° =  anterior (A), 90° =  ventral (V), 180° =  posterior (P), 270° =  dorsal (D); *transverse*: 0° =  dorsal (D), 90° =  left (L), 180° =  ventral (V), 270° =  right (R).

**Figure 4:**
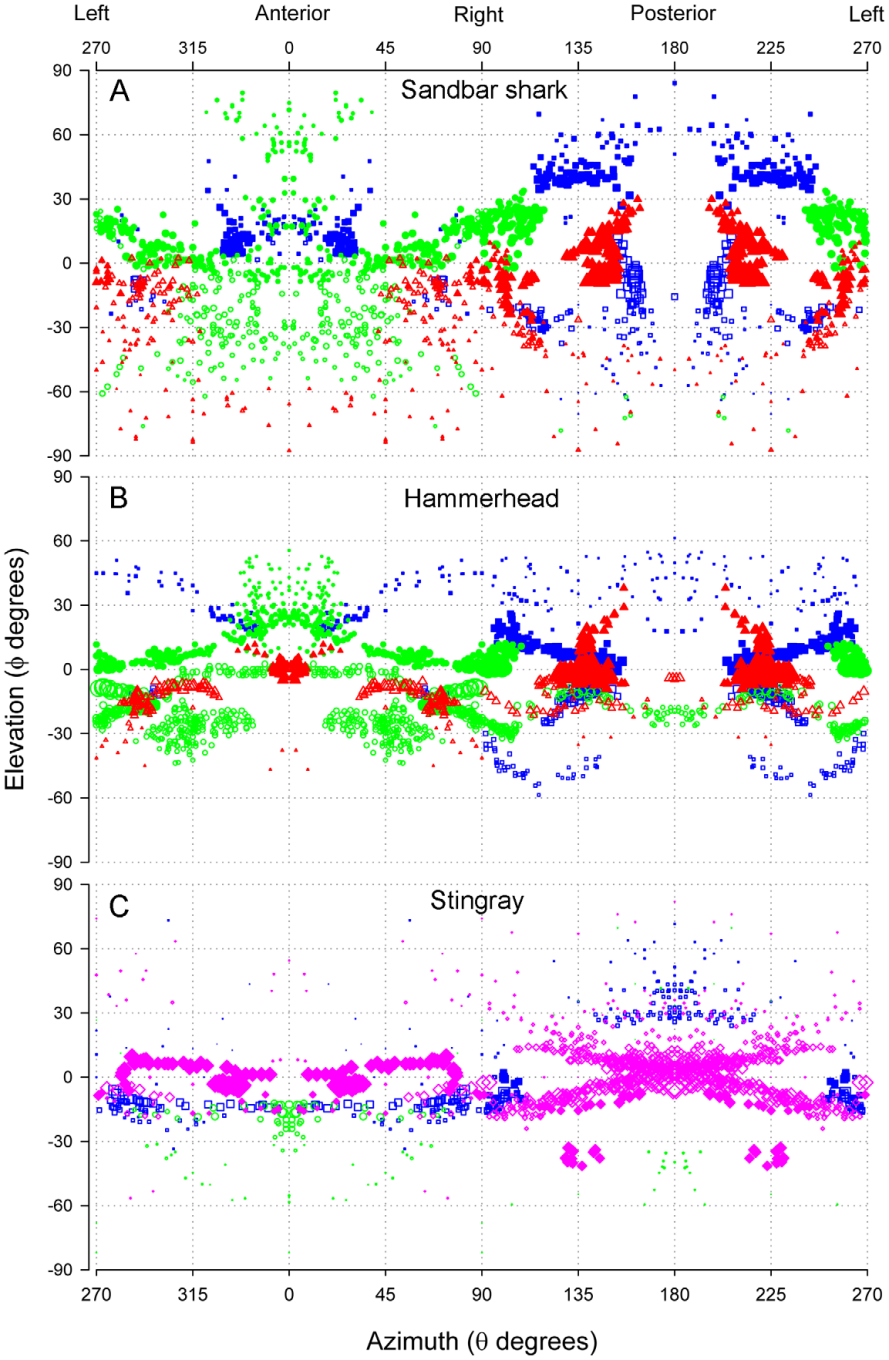
Spherical projections for ampullary groups in the sandbar shark, hammerhead shark and brown stingray. Azimuth (θ degrees) is plotted on the X axis and elevation (ϕ degrees) plotted on the Y axis. The coordinate of 0° θ, 0° ϕ corresponds to the longitudinal axis in the horizontal plane of the animal. Values of θ between 270° and 90° represent canals that project rostrally. Negative values of ϕ indicate canals that project ventrally and positive values of ϕ canals that project dorsally. Ampullary groups are represented by different colors and symbols (BUC  =  blue squares, SOa  =  green circles, SOp  =  red triangles, HYO  =  pink diamonds). Mandibular ampullae are not shown. Symbol size corresponds to relative canal length (larger symbols  =  longer canals).

Each BUC cluster in the sandbar shark is located lateral on the head with several pores that surround the eye (Fig. 2A). A total of 281 canals were counted on the left cluster of which 41% project ventrally and 59% dorsally (Table 1). Dorsal BUC canals averaged 4.55 cm in length and were divided into two subpopulations of canals based on their projection vectors. The first dorsal group showed strong projections towards the tip of the snout at ±330° *alpha* (Fig. 3, horizontal plane) and a vertical elevation centered around 10° θ (Fig. 4). The second dorsal population projects medially and posterolaterally towards the top of the chondrocranium (∼135° *alpha* on the horizontal plane, Fig. 3; 225° θ, Fig. 4) and contains the longest canals with vertical projections (225° *alpha* on the sagittal plane, Fig. 3; 40° ϕ, Fig. 4). The ventral BUC cluster contains the longest canals of the BUC group at 11.7 cm with mean length (4.62 cm) similar to that of the dorsal BUC group. These canals project caudally in the horizontal plane (∼165° *alpha*, Fig. 3). The overall range of elevation for the entire BUC cluster was from −71° to 84° ϕ (Fig. 4).

The SOa was the most rostral cluster in *C. plumbeus* (Fig. 2). The SOa ampullae were arranged in a spherical cluster with their pores located along the anterior edge of the rostrum. This cluster had the highest number of canals (427) with 37% projecting to pores on the dorsal and 63% on the ventral surfaces (Table 1). Dorsal and ventral mean length for the SOa was 4.23 and 3.52 cm, respectively. Both dorsal and ventral subclusters have strong rostral projections of very short canals (θ<180°) with vertical elevations from −78° to +79° ϕ (Fig. 4). The longest SOa canals project primarily in the lateral direction (∼90° *alpha* in the horizontal and transverse planes, Fig. 3).

The left SOp subcluster had a total of 307 canals with 55% projecting to pores on the dorsal and 45% to the ventral surface (Table 1). The sandbar shark SOp ampullae were arranged in a discrete spherical cluster. Dorsal SOp ampullae presented the longest canals with maximum length of 16.11 cm and mean of 8.31 cm. One group of dorsal SOp canals projects laterally toward pores located around the eye (90° *alpha*, Fig. 3). A second group of canals project posterior-laterally towards 150° *alpha* (Fig. 3) with vertical elevation below 30° ϕ (Fig. 4). Elevations for other shorter canals were primarily downward up to −87° ϕ for canals projecting rostrally (θ<180°) and caudally (θ>180°) (Fig. 4).

The sandbar MAN cluster contained 6 canals on the left side of the head with pores positioned caudal to the edge of the lower jaw. Canal length ranged from 3.92 cm to 8.44 cm with a mean canal length of 6.27 cm. These canals projected posterolaterally and were essentially horizontal from −3° to 0° ϕ (Fig. 4).

#### Hammerhead shark

**Figure 5:**
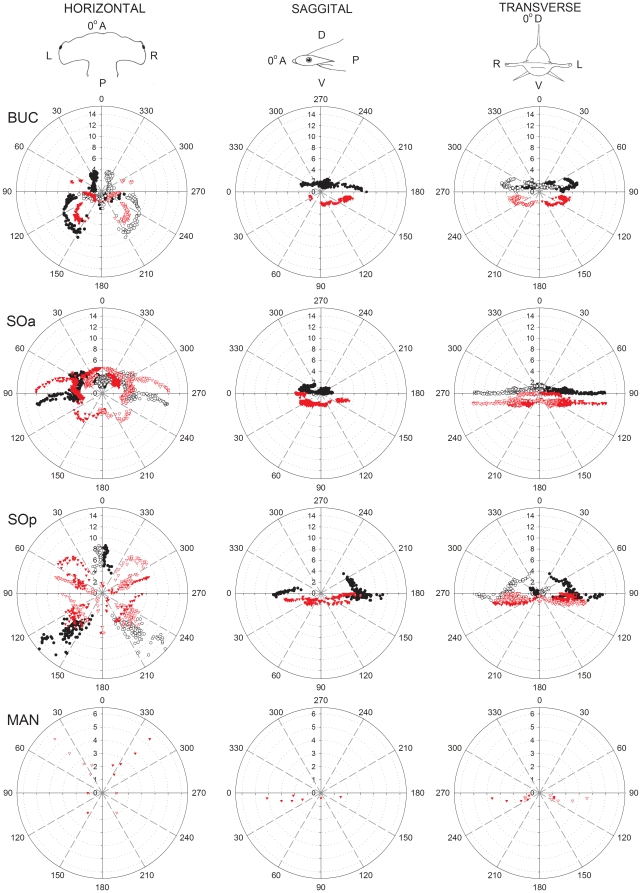
Polar plots of canal projections in three planes of the electrosensory ampullary array of the scalloped hammerhead shark, *Sphyrna lewini*. Plots show canal length (cm) vs. canal orientation angle in the horizontal, sagittal and transverse planes for each ampulla group (BUC  =  buccal; SOa  =  superficial ophthalmic anterior; SOp  =  superficial ophthalmic posterior; MAN  =  mandibular). Projections of ventral and dorsal canals are indicated with red and black symbols, respectively. Projections are shown for both left (filled symbols) and right (open symbols) ampullary clusters. Reference directions for each plane are *horizontal*: 0° =  anterior (A), 90° =  left (L), 180° =  posterior (P), 270° =  right (R); *sagittal*: 0° =  anterior (A), 90° =  ventral (V), 180° =  posterior (P), 270° =  dorsal (D); *transverse*: 0° =  dorsal (D), 90° =  left (L), 180° =  ventral (V), 270° =  right (R).

The hammerhead shark shares common clusters with the sandbar shark and has distinct projections in dorsal and ventral surfaces (see Movie S1). The BUC cluster is located on the lateral aspect of the cephalofoil, medial to the eye and caudal to the nares (Fig. 2B). A left BUC cluster contained 297 canals of which 37% project to dorsal and 63% project to ventral pores (Table 1). Canal lengths for the BUC included the shortest found in this species (1.10 cm) and up to 9.30 cm. Each BUC cluster has dorsal canals that sweep in a 270° arc in the horizontal plane from the anterior to lateral to posterior to medial directions (Fig. 5). Longest dorsal canals are in the posterior lateral direction at an azimuth of 135° *alpha* in the horizontal plane (Fig. 5). Ventral projections show a similar prominent posterior lateral projection but with shorter canals. In total, the BUC dorsal projections cover a near 360° azimuth with maximum elevation near 60° ϕ and a dip to below 30° ϕ in the anterior direction (Fig. 4). Ventral BUC canals are relatively short, have restricted posterior lateral projections and show maximum elevation near −60° ϕ (Fig. 4).

As in the sandbar shark, the hammerhead SO cluster was physically divided into SOa and SOp (Fig. 2B). This separation was enhanced by the lateral rostral cartilage, which provides most of the support for the cephalofoil. The SOa subcluster was located on the leading edge of the cephalofoil with most pores located near the anterior margin. The SOa contained the greatest number of ampullae in any group (774) with 40% projecting to dorsal and 60% to ventral pores (Table 1). Dorsal and ventral SOa canal lengths were similar, ranged from 1.82–12.15 cm with a mean length of approximately 4.5 cm and showed longest canals in the lateral direction. Individual clusters projected across anterior to lateral directions with the ventral cluster extending in medial and posterior directions. Spherical coordinates show that SOa canals that project caudally had weak elevation (−33° to 12° ϕ), whereas SOa canals that project rostrally show a greater vertical projection range (−44° to 56° ϕ) (Fig. 4).

The SOp subcluster was also subdivided by the lateral rostral cartilage into ventral and dorsal ampullary groups. Both ventral and dorsal SOp ampullae were organized in a linear pattern that traveled laterally along a fissure on the lateral rostral cartilage. This linear attenuated morphology is distinct from other *S. lewini* and *C. plumbeus* clusters that show a typical spherical grouped form. There were a total of 284 ampullae in the left SOp with 41% that project to dorsal and 59% that project to ventral pores (Table 1). The dorsal SOp had the longest canal (15.23 cm) and highest mean length of 8.63 cm. Like the sandbar shark, long SOp canals in the hammerhead showed strong posterior projections but differed with direct anterior projections in the horizontal plane. Vertical projections for the SOp ranged from −47° to 38° ϕ but the longest dorsal canals were largely confined to the horizontal plane (−10° to 10° ϕ) (Fig. 4).

The left MAN cluster included 7 ampullae that are located together ventral to the corner of the mouth. The longest canals project anterior towards the center of the mouth with shorter canals projecting lateral and posterior. Canal length range is 1.2–5.5 cm with mean length of 2.7 cm. Vertical projections for these canals were downward from −20° to −4° ϕ (Fig. 4).

#### Brown stingray

**Figure 6:**
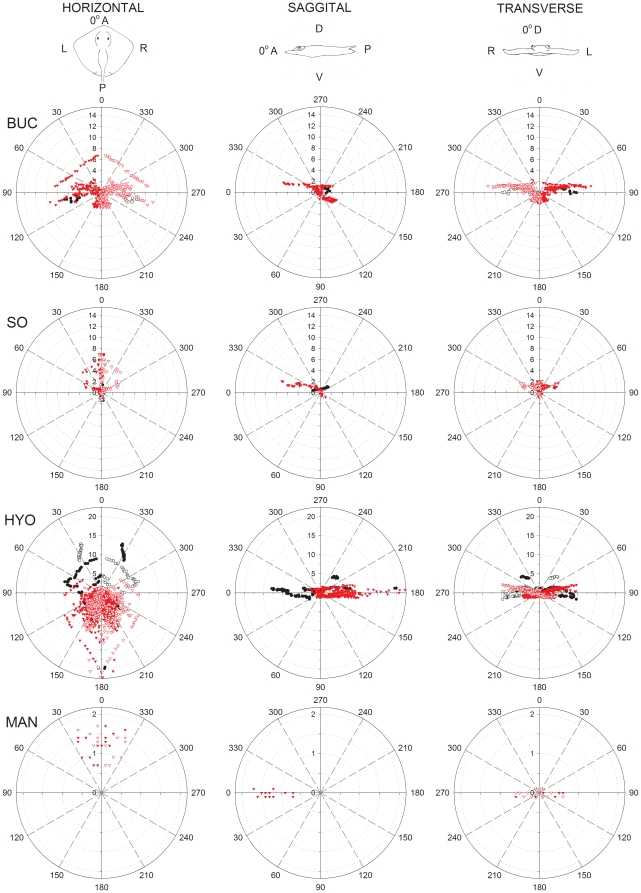
Polar plots of canal projections in three planes of the electrosensory ampullary array of the brown stingray, *Dasyatis lata*. Plots show canal length (cm) vs. canal orientation angle in the horizontal, sagittal and transverse planes for each ampulla group (BUC  =  buccal; SO  =  superficial ophthalmic; Hyo  =  hyoid; MAN  =  mandibular). Projections of ventral and dorsal canals are indicated with red and black symbols, respectively. Projections are shown for both left (filled symbols) and right (open symbols) ampullary clusters. Reference directions for each plane are *horizontal*: 0° =  anterior (A), 90° =  left (L), 180° =  posterior (P), 270° =  right (R); *sagittal* : 0° =  anterior (A), 90° =  ventral (V), 180° =  posterior (P), 270° =  dorsal (D); *transverse*: 0° =  dorsal (D), 90° =  left (L), 180° =  ventral (V), 270° =  right (R).

The stingray body is dorsoventrally flattened and shows distinct differences from either shark species. The stingray BUC clusters are located near to the rostral chondrocranium and below the eye. The left BUC cluster contained 189 canals of which 94% projected to the ventral surface (Table 1). Dorsal canals in the left cluster ranged from 4.17–6.80 cm with a mean length of 5.29 cm, while ventral canals were shorter. The dorsal canals showed minimal vertical elevations and projected laterally at approximately 90° *alpha* in the horizontal and transverse plane (Fig. 6) to pores scattered between two groups of HYO pores. On the ventral surface canals project across a 180° arc from anterior to posterior, with the majority of pores located anterior to the mouth and medial to the nasal capsule (Fig. 2C). The longest canals in the BUC cluster project rostral and lateral on the body. A small subgroup of short canals projected caudally with an elevation of around 30° ϕ (Fig. 4). The elevations of the longest BUC canals were concentrated between 0 and −10° ϕ with only very short canals extending to 60° ϕ (Fig. 4).

The stingray SO cluster is not subdivided as in the two shark species, contains relatively few canals and is located rostrally near the midline, anterior to the BUC cluster (Fig. 2C). Of the 71 canals, 23% project dorsally and 77% ventrally (Table 1). The canals of the SO are among the shortest especially for the dorsal projections that range from 0.71–1.75 cm (mean  = 1.19 cm). Short dorsal canals project almost directly posterior. The longest ventral SO canals project primarily in the horizontal plane (<30°) and to pores on the medial and lateral rostrum (0–60° *alpha* in the horizontal plane) (Fig. 6). There is minimal lateral projection and the few canals that project posterior on the ventral surface are short, <1 cm (Fig. 6, sagittal plane).

The stingray HYO cluster is located ventral to the spiracle, anterior to the gill chamber and slightly posterior of the cranial ridge. It is the largest cluster in the stingray with 467 canals (63% of all canals) of which 17% project to pores on the dorsal and 83% to the ventral surface (Table 1). Dorsal HYO canals had the longest average length (mean  = 8.32 cm) while ventral canals a wider distribution from 0.14–22.22 cm. Combined projections of both left and right HYO clusters show that long canals project omnidirectional in the horizontal plane with the longest ventral canals projecting posteriorly (180°) towards the posterior margin of the disk (Fig. 4, 6.). Other, shorter canal subgroups had projections toward the lateral disc margin (120–160° *alpha* in the horizontal plane) and posteromedially to the superior surface of the chondrocranium (235° in the sagittal plane, Fig. 6). Ventral HYO canals projected mainly towards the posterior of the animal (θ>180°; Fig. 4). Vertical elevation was weak in rostral oriented HYO canals (θ<180°; Fig. 4) and include the longest canals in the array (∼22 cm). There was also a large population of canals that project posterior to pores medial to the gill slits (135–180° *alpha* in the horizontal plane, Fig. 6).

The stingray MAN cluster is located behind the posterior margin of the mouth. The left cluster contained 16 ampullae located in a tight group with canals that range in length from 0.7–1.7 cm (mean  = 1.25 cm). All canals project anteriorly and horizontally to pores along the posterior margin of the lower jaw.

### Standardized canal lengths

**Figure 7:**
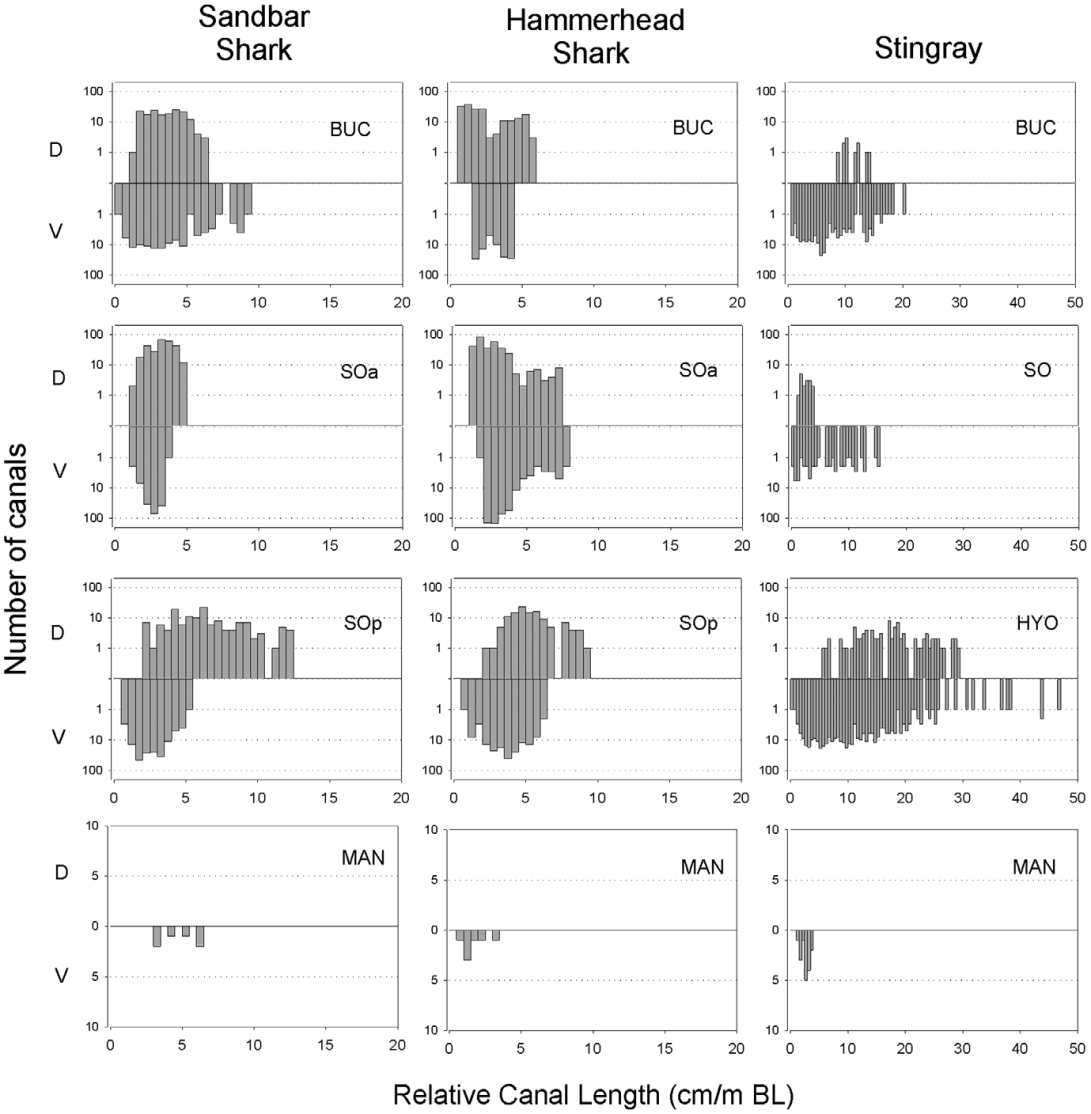
Ampullary canal lengths normalized to body size in the sandbar shark, scalloped hammerhead and brown stingray. Canal lengths were normalized by dividing absolute canal length (cm) by body length (m) (BL =  precaudal length for sharks and disk length for the stingray). Ampullary cluster are BUC  =  buccal, SOa  =  superficial ophthalmic anterior, SOp  =  superficial ophthalmic posterior, HYO  =  hyoid, MAN  =  mandibular. Dorsal (D) and ventral (V) canals for each cluster are shown above and below the zero line, respectively. Bins  =  0.5 cm canal/m BL.

Canal lengths corrected for body size show several similarities and differences in size distributions among ampullary groups and species (Fig. 7). The stingray maximum relative canal length was greater in the BUC (20 cm/BL m) and SO clusters (15 cm/BL m) and showed broader size distributions on the ventral surface compared to either shark species. Relative canal length was greatest in the dorsal SOp for both shark species and in the ventral HYO of the stingray (47 cm/BL m). Among sharks the longest SOp canals project to the dorsal surface whereas dorsal SO canals were relatively short in the stingray. The longest relative MAN canal length was similar for all species at about 5 cm/BL m.

## Discussion

This study presents a detailed analysis of the spatial arrangement and spherical projections of the electrosensory array in three species of elasmobranch fish with different head and body shapes. Below we interpret these data in relation to relevant behavioral contexts and stimulus features in order to assess potential functions for each cluster. Additionally, we use these data to compare the spatial projections of individual clusters among these species and examine for functional patterns of canal convergence, divergence and complementarity. We also discuss the constraint of a dorsoventrally flattened morphology of the hammerhead and stingray on sampling of the vertical electric environment. Finally, we analyze the array morphology in terms of sensory directionality and propose that each ampullary array forms an asymmetrical directional antenna that may compare intensity differences to encode directional information from an electric stimulus.

### Behavioral Contexts, Stimulus Features and Ampullae Cluster Functions

Behavioral and modeling studies show that electroreception is used by sharks and rays for detection of prey, mates, and predators, as well as possible geomagnetic movements [20], [21], [23], [24] that can be categorized by the electric field associated with each stimulus type. Prey, predators and mates produce polar electric fields that are functionally *dipole* in nature, whereas cues that may be used for geomagnetic navigation may resemble *uniform* (passive mode) or whole body (active mode) fields. Sensitivity of ampullary receptors is greatest when a *uniform* electric field is parallel to the canal projection and increases with canal length [18], [20], [40]–[42]. Since ambient uniform fields are weaker in strength than dipole fields at close range [20], an ampullary cluster with long canals will be more sensitive to weak large scale fields than a cluster with short canals and the same projection vectors. Conversely, a cluster with numerous short canals will be suitable for detection of strong fields from cryptic prey at close range since their primary afferent neuron discharges saturate at higher intensities [18].

The complexity of the electrosensory array morphology, receptor response properties and relevant electric fields indicate several modes of electrosense function in a sea water environment. The non-trivial resistance of the skin in marine elasmobranchs was shown in the thornback ray to limit the penetration of local (i.e. polar) electric fields that makes the voltage drop across the skin the effective stimulus for ampullae with both short and long canals (20). Directional information for such polar electric fields may be derived from somatotopically and centrally mapped skin pores that detect local field perturbations, as occurs for microampullae electroreceptors in freshwater rays and tuberous electroreceptors in electric teleosts. In contrast, detection of larger scale fields that span and invade the body (e.g. external uniform fields) may be enhanced by long canal length and internal voltage gradients. The higher sensitivity of ampullae with long canals to weak uniform fields provides enhanced detection of a changing field and potential directional information if their outputs are directionally mapped or processed centrally. In addition, ampullae that share a common cluster reduce self-generated internal noise by common-mode rejection in the brainstem that ultimately enhances signal to noise ratio [20], [43], [44].

The SOp ampullary clusters contain the longest canals in both shark species. In the hammerhead shark the SOp is separated into distinct dorsal and ventral subgroups with the ampullae of both organized into a linear rather than typical spherical cluster. This linear arrangement results from the lateral expansion of the cephalofoil. In the hammerhead shark 75% of the dorsal SOp subgroup canals project exclusively in the posterior-lateral quadrants, while the remaining project anteriorly (Fig. 4, Table 2). The elevations of these long canals are aligned near 0° relative to the rostrocaudal axis, and indicate best sensitivity to weak fields in the horizontal plane of the body in the forward and posterior-lateral directions. In the sandbar shark, almost half of the dorsal SOp also projects to the posterior-lateral quadrant, while the remainder project nearly lateral. Since these long canals are associated with the most sensitive ampullae and lack significant elevation, the dorsal SOp group in both shark species should be the most sensitive to geomagnetic induced uniform horizontal electric fields associated with ocean current flows and the passive mode of electro-orientation.

**Table 2:** Direction and elevations of electrosensory canal projections for the electrosensory ampullary clusters of the sandbar shark (*Carcharhinus plumbeus*), scalloped hammerhead shark (*Sphyrna lewini*) and brown stingray (*Dasyatis lata*).

	Sandbar Shark			Hammerhead			Stingray		
Cluster	Primary Projection	Elevation		Primary Projection	Elevation		Primary Projection	Elevation	
		D	V		D	V		D	V
BUC	AP	+++	++	PL	++	++	AL	++	+
SOa	L	+++	++	L	++	+	A*	++	+
SOp	PL	+	+++	ALP	+	+			
HYO	*np*			*np*			ALP	++	+
MAN	PL	*np*	0	AM	*np*	+	A	*np*	0

Primary projections indicate directions of the longest, most sensitive canals. Elevation indicates maximum vertical projection range for the majority of canals in each cluster: ±10° (0), 0–30° (+), 0–60° (++) and 0–90° (+++). BUC  =  buccal, HYO  =  hyoid,

SOa  =  superficial ophthalmic anterior, SOp  =  superficial ophthalmic posterior. The HYO is not present (*np*) in these shark species.

*The small stingray superficial ophthalmic cluster is not subdivided to anterior and posterior subgroups. Directions are A  =  anterior, L  =  lateral, P  =  posterior, M  =  medial, V  =  ventral projection, D  =  dorsal projection.

The SOa cluster has the greatest number of ampullae in these shark species. All of the pores are located along the anterior edge of the rostrum and rostral to the mouth. Canal lengths are short when compared to the SOp cluster. This arrangement may be best for detection of strong dipole fields along the leading edge of the rostrum, such as those produced by nearby prey, mates or predators. In total, there are >600 more canals in the hammerhead than sandbar shark, due largely to a greater number of SOa canals in the former species. This increase in canal number maintains a similar pore density with a larger head surface area, as reported previously [34]. However, at larger distances from the source the dipole field may approximate that of a uniform field, thus longer and more sensitive lateral projecting canals of the SOp (and others) may also be important in detection of dipole fields.

In the stingray, the SO cluster differs from these sharks in that it is much less subdivided and contains relatively few canals. However, the stingray's SO cluster is similar as it contains predominantly short canals (some of the shortest relative canal lengths measured), indicating that its function may be best suited for detection and localization of small dipole prey near the snout. Additionally, the short canals could provide information about charge separation across the fish body as induced by movement through the Earth's magnetic field [26], [27] but would require relatively strong potentials. As in the shark, stingray SO canals project primarily in anterior and posterior ventral directions. The ventral projections of the SO cluster consist of short to moderately long canals, with the longest canals projecting anteriorly to pores located along the midline of the rostrum (Table 2). A few of the shorter canals (<4 cm) project anterolateral in the horizontal plane while the shortest canals (<1 cm) project posteriorly. The majority – and longest – of the stingray SO canals have sagittal vectors of 10°–30° (Fig. 6), with little vertical component (<1 cm) thus are relatively insensitive to weak vertical fields. This is consistent with behaviors where stingrays probe the benthic substrate to locate buried prey or mates that are near to the snout.

The approach of juvenile scalloped hammerhead sharks to a dipole often consists of a series of C-shape turns, where the closest side of the cephalofoil to the dipole functioned as a pivot and remained stationary in relation to the center of the dipole [30]. This behavior may be mediated by the BUC cluster which is located near the lateral edge of the head and has canals that radiate over a ¾ semicircle arc (Fig. 2). In contrast, the BUC cluster of the stingray is not located laterally, but more medial and anterior to the chondrocranium. Long dorsal canals project strongly in the horizontal plane toward the lateral margin of the disk. The ventral BUC contains considerably more canals that project across a 180° arc in the anterior, lateral and posterior directions. The majority of these pores are found anterior to the mouth and medial to the nasal capsule, but a subpopulation of longer canals project to the disc margin. Thus in the stingray, the BUC function in prey detection and capture may have shifted to regions near the mouth rather than lateral on the head as found in the shark. Future experiments in which specific ampullary subgroups are inactivated are needed to test ampullary group function in the detection and orientation to dipole and uniform electric fields.

Of the three species examined in this study, only the stingray has an HYO cluster. The HYO is found in more basal sharks such as *Squalus* but is lost in the more derived carcharhiniform sharks. Like the shark SOp, the stingray HYO contained the longest and greatest number of canals. Dorsally, canals projected primarily to pores along the circumference of the disc, as well as to the rostrum and superior chondrocranium. Due to the dorsoventral compression of the body, only the canals projecting to the apex of the fish's head had significant vertical components. The long HYO canals project to the caudal margins of the disc and would be ideal for detection of geomagnetic induced uniform electric fields in the passive mode of orientation [26], [40], or also weak dipole fields from prey as recently modeled in the skate [32]. Thus, the stingray HYO may have subsumed the detection of weak uniform fields in the horizontal plane that is possibly served by the SOp in these sharks. Also, unlike other clusters in the stingray that project primarily laterally, the most numerous projections of the HYO are medial. A large number of these canals project to pores medial to the gill chamber, indicating a possible function of this subgroup is to provide higher electrosensory brain nuclei with information used in common-mode rejection of the animal's own ventilatory movements [45]. In addition, vertical canals in the HYO group were proposed candidates for detection of vertical fields used for a geomagnetic compass sense in other elasmobranchs [27] possibly indicating divergent functions for this group in pelagic species. In wide-ranging sharks that lack hyoid ampullae, distinct vertical projections of BUC canals exist for the sandbar and hammerhead sharks (this study) and in the white shark (40). Thus, a geomagnetic compass sense due to swimming-induced motion may be mediated by different ampullary clusters or cluster subgroups among taxa.

The restricted location and limited number of MAN canals in all three species (immediately posterior to the lower jaw) indicates a probable function to stimulate mouth opening during feeding strikes. The bilateral, anterior and medial projections of these canals indicate a conserved relative length and function that may include alignment of small dipoles near the tip of the mouth. Some bilateral stimulation of the MAN likely occurs when male sharks and rays approach to bite the female pectoral fins or body during courtship and copulation [23], [46]–[48].

### Functional Convergence, Divergence and Complementarity among Ampullary Clusters

Our analysis demonstrates that ampullary clusters differ in their numbers, proportions of long and short, and projection vectors of canals, but that considerable overlap also exists. We report evidence of convergent and complementary projections among clusters and canal subgroups within a single species that may provide information important for signal processing in the brain that is consistent with the functional subunit hypothesis [40]. For example, there were strong lateral projections by canals in both the BUC and HYO clusters of the stingray, and to the posterior lateral quadrant by both the BUC and SOp in the shark. Although it is not known whether central projections of axons from these different cluster subgroups converge on single principal cells in the dorsal octaval nucleus, each nerve ramus does project to a distinct region and retains somatotopic organization in the brainstem relative to pore position on the skin [47]. Convergence of primary afferent neurons from these different canal subgroups with common (or oppositely oriented) projection vectors upon common principal cells could enhance directional sensitivity to electric fields. Similarly, complementary directional projections from canals in different clusters may be integrated centrally to provide omnidirectional information during processing of electric information. In the horizontal plane, directional complementarity is seen in the ventral BUC and ventral HYO of the stingray, and in the dorsal BUC, SOa and SOp of the hammerhead and sandbar sharks. Neurophysiological experiments that test directional input of canals with common and complementary projections to neurons in the central nervous system are needed to determine central processing for these canal groups. It would also be worthwhile to define the ascending connections of the different subdivisions of the dorsal nucleus to see if these are consistent with control of distinct behavioral outputs.

Although a number of past studies have addressed the morphology of the electrosensory system in elasmobranchs, most only reported pore counts and two-dimensional, qualitative line drawings of the ampullary canals [33], [36], [49]. A previous study [40] recognized the potential importance of canal vector (length and projection angle) for defining directional electrosensory sensitivity and noted that the dorsoventrally flattened batoids have very short canals that project in the vertical plane. Our spherical coordinate analysis clearly demonstrates that the projection of long canals in the dorsoventrally flattened body of the stingray and also the dorsoventrally flattened head of the hammerhead shark are limited to a narrower elevation when compared to those in the conical head of the sandbar shark. While the sandbar shark is able to sample most of the vertical plane (up to ± 90° vertical, Fig. 4), the hammerhead shark only samples about 2/3 of that plane (±60° vertical, Fig. 4). The longer canals in the stingray are confined to narrow band in the vertical (±30°, Fig. 4) with the exception of the long posterior-lateral canals at ∼ −40° elevation. The behavioral implications of these morphological constraints may be relevant to the use of active electro-orientation/navigation [20], [25], [27] among different species. Animals swimming through the Earth's magnetic field will generate an electric field across the head and body that is orthogonal to the magnetic field and swimming direction. These potentials will be dorsal-negative when swimming eastward and dorsal-positive when moving westward with no voltage difference when swimming parallel to the horizontal magnetic field (north or south). By comparing the voltage difference between canals in the dorsal and ventral surfaces elasmobranchs could discriminate their general compass heading (eastward vs. westward). The dorsoventrally flattened hammerhead sharks and brown stingray should be less sensitive to vertical induced electric fields near the magnetic equator than at higher latitudes. However, the caveat remains that all species which have short canals with strong vertical projections (Fig. 4, Table 2) may still detect strong induced fields during locomotion. For example, a shark swimming at only 5 cm/sec in a magnetic field of 0.5 G will induce a threshold electric field of about 5 ηV/cm [26] that could be detected by populations of ampullae with short but vertical canals. Future experiments are needed to determine whether directional information from geomagnetic induced electric stimuli are enhanced by ampullary arrays that consist of canals with different sensitivities and spatial projections.

After correction for body size, the laterally expanded cephalofoil of the hammerhead shark does not show longer canals in the BUC or SOp than the sandbar shark, but does show more and longer canals in the SOa group (Fig. 7). Although, SOa mean relative canal lengths are similar for both species the range in the hammerhead is twice that of the sandbar shark. This difference in canal length range is apparently due to a greater number of canals longer than 5 cm/BL m. Additionally, the scalloped hammerhead shark's longest canals are confined to a narrow 20° band around the horizontal plane (−10° to 10° θ) (Fig. 4). Thus, sensitivity to horizontal fields may have increased in *S. lewini* as a result of the lateral expansion of the cephalofoil. This may make the animal highly sensitive to horizontal fields induced by the movement of water currents (passive mode) through the vertical component of the Earth's magnetic field as shown for stingrays [26].

There is empirical evidence that confirms higher sensitivity of longer canals to *uniform* electric fields [42]. However, although modeled [32], a relationship between length and sensitivity has not been reported for *dipole* fields. It was recently proposed that the electrosensory hydrogel and individual canal morphology promote the potential difference between each ampullary chamber and its corresponding pore [12], [13], [50]. Accordingly, longer canals have higher resistance and create a larger potential difference, thereby increasing the sensitivity of longer canals to any type of electric field. Our canal length data show that the hammerhead shark has a greater number of long canals than the sandbar shark, and implies that the cephalofoil provides higher relative sensitivity to electric fields when compared to a similar size shark with a pointed rostrum. The expanded lateral arrangement of canals in the hammerhead shark is proposed to enhance angular resolution and approach to a dipole source (50). However, in experiments that compared behavioral responses of juvenile hammerhead and sandbar sharks to dipole electric fields, no such sensitivity difference (orientation from greater distances) was observed [30]. Since the animals used for that behavioral test were juveniles, it is possible that in juveniles the canal length difference between both species is not large enough to convey an advantage to hammerhead sharks for detection of dipole prey at larger distances. As both species grow in size the relative canal lengths will remain approximately the same but the absolute canal length difference will increase in magnitude signifying an increase in sensitivity. To answer these questions additional experiments that compare neural responses from adult canals of known length to controlled dipole fields are needed.

### Target Detection and Direction

The directional sense of electroreceptor systems were studied in gymnotid electric fishes and the paddlefish. Electric fish are unable to determine the location of an electric source instantaneously without feedback. Instead, they locate an electric source by continuously sampling and following electric field lines [10], [51]. In the paddlefish, electroreceptors receive enough voltage information in the time domain for cells in the dorsal nucleus to compute a time derivative used for target localization [52], [53]. In sharks, Kalmijn [29] proposed two mechanisms that could be used to locate electric field sources. Similar to electric fish, sharks could follow electric field lines that would result in a spiral approach path towards the source. Alternatively, sampling of the stimulus across the electrosensory array could mediate a direct turn towards the source. Behavior studies [30] showed that juvenile hammerhead sharks made a direct turn towards an electric source 96% of the time they were presented with a dipole electric stimulus. This indicates that sharks may be able to rapidly derive the location of an electric source without having to follow electric field lines in all cases. More work is needed to determine the time delay between detection of and orientation to electric field sources to resolve this question.

This study shows that sharks and rays have a peripheral electrosensory array system that is directionally sensitive and may be used to mediate orientation and target localization behaviors in three dimensions. Barn owls can locate a sound source using two ear receivers without successive approximation by determining target elevation via processing of stimulus intensity differences and azimuth by stimulus time delay in the midbrain [54]–[56]. It is possible that elasmobranchs can compare intensity differences across individual vector elements of the electrosensory array to derive the direction or location of an electric field through the use of azimuth and elevation information from the stimulus. Feature detection may also be enhanced by the asymmetry of the ampullary array which can enhance directional sensitivity across time [32].

## Materials and Methods

### Ethics Statement

All fishing and husbandry were approved by the University of Hawaii's Institutional Animal Care and Use Committee (IACUC) for protocol 01-042-04.

### Study Specimens

Large adult specimens of all study species were used for measurement of canal projections. Kajiura [31] previously demonstrated that the number of ampullary canals and pore distributions in *S. lewini* and *C. plumbeus* does not change with size (age). One male *S. lewini* (precaudal length  = 1.61 m, standard length  = 1.76 m and total length  = 2.31 m) and one male *C. plumbeus* (precaudal length  = 1.30 m, standard length  = 1.45 m and total length  = 1.79 m) were captured on a longline and euthanized. Their heads were fixed in 4% formaldehyde for 4 weeks, rinsed with freshwater for 36 hours and preserved in 50% isopropanol. A female brown stingray (*D. lata*) was captured via handline from Kaneohe Bay, euthanized in MS-222, and fixed in 4% formaldehyde for one week. The specimen (total length  = 1.28 m, dorsal length  = 0.475 m, ventral length  = 0.41 m, disk width  = 0.55 m) was rinsed with fresh water for 48 hours and stored in 50% isopropanol.

Juvenile hammerhead and sandbar sharks were used to determine swimming inclinations for head alignment during canal measurements. Juvenile sharks were captured by handline, transported in a circular 1-m diameter hemisphere by boat to the laboratory, and released in an 8-m diameter tank with a flow through seawater system. Sharks were fed frozen fish and squid once a week. All fishing and husbandry were approved by the University of Hawaii's Institutional Animal Care and Use Committee (IACUC) for protocol 01-042-04.

### Ampullary array measurements

Spatial locations of ampullary canals were estimated by measurement of coordinates for each electrosensory pore and its corresponding ampulla relative to the midline longitudinal body axis. Wooden stereotactic box frames were constructed for each species to the precise dimensions of the head (and body for the ray). Each frame was fitted with rulers on the transverse (X) and longitudinal (Y) axes of the horizontal plane. A t-square was used to align values on each ruler with a desired ampullary pore. A caliper was placed on the t-square, directly over each pore, and the depth probe on the bottom of the caliper was used to obtain vertical (Z) coordinates. All measurements were made to the nearest millimeter. To eliminate error due to repositioning of the heads, coordinates for six landmarks on each specimen were recorded. These landmarks were used to position the head prior to any measurements or dissection.

Shark specimens were positioned in each frame with their heads at normal swimming inclination relative to the horizontal plane. To determine normal head inclination, a digital camera (Canon PowerShot S45) in an underwater housing was mounted horizontally on a wood stand 30 cm above the bottom in the center of a large circular tank in which juvenile sharks swam freely about. High resolution images (4.0 megapixels) were taken of the head of free-swimming sharks of each species (n = 3 per species). Head inclination relative to horizontal plane was calculated from digital images with SigmaScan Pro 5. For *C. plumbeus* natural head angle while swimming was measured as 2° rostrum-up inclination of the line connecting the tip of the rostrum with the center of the eye relative to the horizontal plane. For *S. lewini* there is a 3° rostrum-up inclination of the cephalofoil plane (the line connecting the tip of the rostrum and the caudal edge of the cephalofoil) relative to the horizontal plane. Due to the demersal nature and dorsoventral compression of the stingray, the effective swimming inclination was assumed to be zero degrees.

A 3D frame model for the surface of the right side of the head was prepared for each species. A 1×1-cm grid was drawn on the surface of the right side of the specimen and x, y, z coordinates were taken at the intersection of grid lines. Digital images were taken of the dorsal and ventral head surfaces and each pore numbered sequentially. For each pore the canal and ampulla were exposed by dissection of the dermis and connective tissues. Coordinates were then taken for the ampullae and corresponding skin pore. Once a pore-ampulla pair was measured, the ampulla, canal and pore were removed. After the completion of the dorsal side each specimen was repositioned ventral-side-up in the stereotactic frame with the appropriate orientation to the horizontal plane (sandbar −2°, hammerhead −3°). The stingray was inverted into a mold form-fitted to the dorsal surface and made from Plaster of Paris. The mold maintained the ventral surface at zero degrees, parallel to the bottom of the jig. The location of pores, canals and ampullae were measured only for the left side of the head. We assumed bilateral symmetry of the electrosensory array [34] and extrapolated our measurements to the right side of the head to obtain vectors for the bilateral array.

### Data analysis

MATLAB (Mathworks Inc., Natick, Massachusetts, USA) was used to generate a 3D wire frame model of the shark's skin with data taken from the right side of each specimen. Ampullary canal spatial maps were produced with the MATLAB ‘plot3’ function for each cluster and were superimposed on the wire frame model. Canal length was plotted against canal orientation for the dorsal and ventral canals of all clusters in all planes (horizontal, sagittal and transverse) using SigmaPlot 11 to produce polar plots for each species. Reference positions for each plane are as follows: horizontal (0° =  anterior, 90° =  left, 180° =  posterior, 270° =  right); sagittal (0° =  anterior, 90° =  ventral, 180° =  posterior, 270° =  dorsal); transverse (0° =  dorsal, 90° =  left, 180° =  ventral, 270° =  right). Cartesian coordinates (x, y, z) for each cluster were converted into polar coordinates (MATLAB ‘cart2pol’ function) for vector analysis. Polar coordinates (canal length, *r*; and projection angle, *alpha*) were analyzed in regards to the horizontal, sagittal and transverse planes of the shark to determine the resultant vector for each cluster. Ampullary cluster resultant vectors in each plane were calculated as mean canal length (*r_avg_*) and mean projection angle (*alpha*) for each cluster. Resultant vector data were plotted into polar plots using MATLAB's ‘compass’ function. Reference positions for each plane are as indicated for the polar plots. A movie that shows rotation of the dorsal and ventral projections was constructed for the hammerhead shark with the MATLAB movie functions.

Canal projections were also analyzed using spherical coordinates, azimuth (θ, *theta)* and elevation (ϕ, *phi*) (Fig. 1B), which allows representation of a three-dimensional environment with a two-coordinate system. This permits the visualization of each individual ampullary canal projection to determine which part of the three-dimensional electrical environment is sampled. Cartesian coordinates were converted into spherical coordinates with MATLAB's ‘cart2sph’ function and graphs were produced with the ‘scatter3’ graphing function. Spherical data were graphed by cluster and canal length. The canal orientations represented with spherical coordinates refers to the spatial vector of the canal in reference to the ampulla, and is not relative to the midline of the fish's body.

To compare canal length (i.e. canal sensitivity) between species, canal length data were normalized by dividing each canal measurement in centimeters by body size in meters (precaudal length for shark species, disk length for the stingray). Normalized canal length data were organized into separate dorsal and ventral histograms for each ampullary group.

## Supporting Information

Movie S1

Spatial projections of ampullary electrosensory canals in the scalloped hammerhead shark, *Sphyrna lewini*. The head is rotated 360 degrees in the horizontal plane with sequential frontal, offset dorsal and direct dorsal views. Canal colors indicate ampullary groups of the buccal (blue), superficial ophthalmic anterior(green), superficial ophthalmic posterior (red) and mandibular (blue green) clusters based upon cranial nerve innervation. Dorsal (D) and ventral (V) canal projections are indicated on opposite sides of the head during rotation.

WMV
